# Prediction of noninvasive ventilation failure using the ROX index in patients with de novo acute respiratory failure

**DOI:** 10.1186/s13613-022-01085-7

**Published:** 2022-12-05

**Authors:** Jun Duan, Juhua Yang, Lei Jiang, Linfu Bai, Wenhui Hu, Weiwei Shu, Ke Wang, Fuxun Yang

**Affiliations:** 1 Department of Respiratory and Critical Care Medicine, The First Affiliated Hospital of Chongqing Medical University, Chongqing, 400016 China; 2 Department of Respiratory and Critical Care Medicine, The Chongqing Western Hospital, Chongqing, 400051 China; 3 Department of Critical Care Medicine, Yongchuan Hospital of Chongqing Medical University, Yongchuan, Chongqing, 402160 China; 4 Department of Respiratory and Critical Care Medicine, The Second Affiliated Hospital of Chongqing Medical University, Chongqing, 400010 China; 5 Department of ICU, Sichuan Provincial People’s Hospital, University of Electronic Science and Technology of China, 32# W. Sec 2, 1st Ring Rd, Chengdu, 610072 China

**Keywords:** Acute respiratory failure, Noninvasive ventilation, ROX index

## Abstract

**Background:**

The ratio of SpO_2_/FiO_2_ to respiratory rate (ROX) index is commonly used to predict the failure of high-flow nasal cannula. However, its predictive power for noninvasive ventilation (NIV) failure is unclear.

**Methods:**

This was a secondary analysis of a multicenter prospective observational study, intended to update risk scoring. Patients with de novo acute respiratory failure were enrolled, but hypercapnic patients were excluded. The ROX index was calculated before treatment and after 1–2, 12, and 24 h NIV. Differences in predictive power for NIV failure using the ROX index, PaO_2_/FiO_2_, and PaO_2_/FiO_2_/respiratory rate were tested.

**Results:**

A total of 1286 patients with de novo acute respiratory failure were enrolled. Of these, 568 (44%) experienced NIV failure. Patients with NIV failure had a lower ROX index than those with NIV success. The rates of NIV failure were 92.3%, 70.5%, 55.3%, 41.1%, 35.1%, and 29.5% in patients with ROX index values calculated before NIV of ≤ 2, 2–4, 4–6, 6–8, 8–10, and > 10, respectively. Similar results were found when the ROX index was assessed after 1–2, 12, and 24 h NIV. The area under the receiver operating characteristics curve was 0.64 (95% CI 0.61–0.67) when the ROX index was used to predict NIV failure before NIV. It increased to 0.71 (95% CI 0.68–0.74), 0.74 (0.71–0.77), and 0.77 (0.74–0.80) after 1–2, 12, and 24 h NIV, respectively. The predictive power for NIV failure was similar for the ROX index and for the PaO_2_/FiO_2_. Likewise, no difference was found between the ROX index and the PaO_2_/FiO_2_/respiratory rate, except at the time point of 1–2 h NIV.

**Conclusions:**

The ROX index has moderate predictive power for NIV failure in patients with de novo acute respiratory failure.

## Background

The use of noninvasive ventilation (NIV) is common in patients with de novo acute respiratory failure [1]. Its use decreases the odds ratio (OR) of intubation relative to conventional oxygen therapy [2]. However, the rate of NIV failure is high in this patient population, ranging from 40% to 65% [3–6]. Furthermore, a two- to six-fold greater rate of mortality is seen in patients with NIV failure relative to that in patients with NIV success [7]. Among patients with NIV failure, delayed intubation further increases the risk of death [8, 9]. Early identification of high-risk patients and early application of intubation is a promising strategy for reducing mortality [10].

The ratio of SpO_2_/FiO_2_ to respiratory rate (ROX) index was developed by Roca et al. [11] to predict the failure of high-flow nasal cannula (HFNC). They showed that the area under the receiver operating characteristics curve (AUC) was between 0.66 and 0.80 from initiation to 24 h HFNC when the ROX index was used to predict HFNC failure [12]. The index has also been used to predict HFNC failure in patients with COVID-19 pneumonia [13, 14]. Because measurement of the ROX index is feasible, effective, and reproducible, it is widely used to predict HFNC failure [15, 16]. To the best of our knowledge, the value of the prediction of NIV failure obtained using the ROX index is unclear. Here, we explored the predictive power of the ROX index for NIV failure in patients with de novo acute respiratory failure.

## Methods

This was a secondary analysis of a multicenter prospective observational study performed to update HACOR scoring [17]. It was conducted in 18 hospitals in China and Turkey from September 2017 to September 2021. The relevant ethics committees approved the study and informed consent was obtained from the patients or their family members. We enrolled patients with de novo acute respiratory failure. However, hypercapnic patients were excluded. De novo acute respiratory failure was defined as occurrence of respiratory failure without chronic respiratory disease, chronic heart disease, asthma, cardiogenic pulmonary edema, cardiac problems other than cardiogenic pulmonary edema, or postoperative hypoxemia [2–4, 17, 18].

Patients who were admitted to the participating centers were managed by the attending physicians, respiratory therapists, and nurses in charge. NIV was used to avert respiratory failure if the respiratory rate (RR) was > 25 breaths/min, if a clinical presentation of breathlessness at rest emerged (such as active contraction of the accessory inspiratory muscles or paradoxical abdominal motion), or PaO_2_ fell to < 60 mmHg at room air pressure or PaO_2_/FiO_2_ fell to < 300 mmHg with supplemental oxygen [17]. The formula of 21 + 4 × flow (L/min) was used to estimate the FiO_2_ if supplemental oxygen was used [19, 20]. A face mask or nasal mask was used to connect the patient to the ventilator. If NIV intolerance occurred, HFNC was used as an alternative strategy to prevent intubation. NIV intolerance was defined as termination of NIV due to discomfort, even in case of intermittent use [21].

We collected diagnoses and underlying diseases at admission. Pneumonia was assessed as new or increasing pulmonary infiltrate in chest radiographs coupled with clinical findings suggesting infection, such as new onset of fever, purulent sputum, cough, chest pain, leukocytosis, decline in oxygenation, and so on [22]. Acute respiratory distress syndrome (ARDS) was diagnosed as follows: (1) presence of acute hypoxemic respiratory failure with PaO_2_/FiO_2_ less than 300 mmHg; (2) within 1 week of a clinical insult or the presence of new (within 7 days) or worsening respiratory symptoms; (3) bilateral opacities in computed tomographic (CT) scans or chest X-rays not fully explained by effusions, lobar or lung collapse, or nodules; and (4) respiratory failure not fully explained by cardiogenic pulmonary edema or fluid overload [23, 24].

Consciousness was assessed using the Glasgow Coma Scale (GCS). GCS, heart rate, RR, blood pressure, pH, PaCO_2_, PaO_2_/FiO_2_, and SpO_2_ were collected before treatment and after 1–2, 12, and 24 h NIV. Disease severity was assessed with the sequential organ failure assessment (SOFA) score. The primary outcome was NIV failure, which was defined as the requirement of intubation [17]. The secondary outcomes were duration of ICU stay and duration of hospital stay.

We used MedCalc (MedCalc Software Ltd, Ostend, Belgium) and SPSS (version 25.0; IBM Corp., Armonk, NY) to analyze the data. Normally distributed continuous variables were analyzed using Student’s *t* test, and abnormally distributed continuous variables were analyzed using the Mann–Whitney *U* test when appropriate. Categorical variables were analyzed using the chi-squared test or Fisher’s exact test, where appropriate. The ability to predict NIV failure was tested with the AUC. The Hanley and McNeil method was used to test the difference in AUC between the ROX index and PaO_2_/FiO_2_ or between the ROX index and PaO_2_/FiO_2_/RR [25]. Three cutoff values were selected for clinical reference at probabilities of NIV failure equal to 25%, 50%, and 75% [26]. Patients with probabilities of NIV failure less than 25%, 25–50%, 50–75%, and more than 75% after 1–2 h NIV were termed the low, moderate, high, and very high risk for NIV failure groups, respectively. A *p* value less than 0.05 was considered to indicate statistical significance.

## Results

**Fig. 1:**
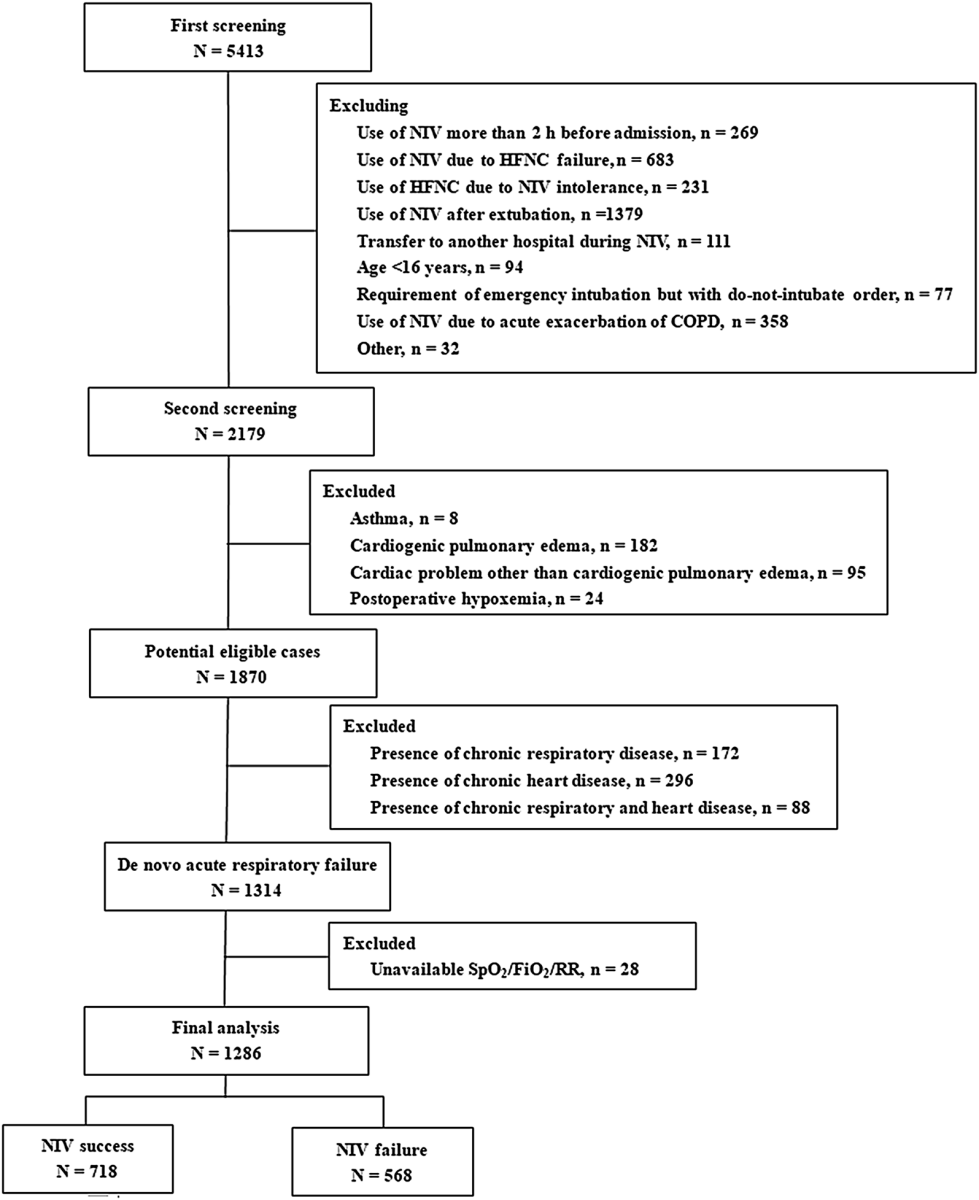
Patient screening. *HFNC* high-flow nasal cannula, *NIV* noninvasive ventilation, *RR* respiratory rate

A total of 5413 patients were screened (Fig. 1). In all, 1286 patients were enrolled in the final analysis. Of these, 23 cases (1.8%) had missing data concerning PaO_2_ at some time point. The rate of NIV failure was 44% (568/1286). Patients with NIV failure had a higher SOFA score (6.1 ± 2.9 vs. 4.8 ± 2.4, *p* < 0.01) than those with NIV success (Table 1). They also had higher hospital mortality than successful patients (43% vs. 4%, *p* < 0.01). The PaO_2_/FiO_2_ values collected before and after 1–2 h NIV were lower in patients with NIV failure than in those with NIV success (145 ± 76 vs. 167 ± 91 mmHg, *p* < 0.01; and 156 ± 82 vs. 213 ± 92 mmHg, *p* < 0.01, respectively). Similar results were found for SpO_2_.

**Table 1:** Comparisons between patients with NIV success and failure

	NIV success *N* = 718	NIV failure *N* = 568	*p*
Age, years	57 ± 17	60 ± 17	< 0.01
Male	430 (60%)	381 (67%)	< 0.01
SOFA score	4.8 ± 2.4	6.1 ± 2.9	< 0.01
Presence of ARDS	173 (24%)	181 (32%)	< 0.01
Use of vasopressor during NIV	69 (10%)	95 (17%)	< 0.01
Diagnosis			
Pneumonia	362 (50%)	409 (72%)	< 0.01
Nonpulmonary sepsis	108 (15%)	81 (14%)	0.75
Pancreatitis	127 (18%)	37 (7%)	< 0.01
Other	121 (17%)	41 (7%)	< 0.01
Underlying disease			
Hypertension	234 (33%)	165 (29%)	0.18
Diabetes mellitus	153 (21%)	106 (19%)	0.26
Chronic kidney disease	65 (9%)	50 (9%)	0.92
Chronic liver disease	28 (4%)	32 (6%)	0.15
Variables collected before NIV			
GCS	14.7 ± 0.8	14.5 ± 1.5	< 0.01
Heart rate, beats/min	114 ± 24	117 ± 24	0.02
Respiratory rate, breaths/min	30 ± 7	33 ± 8	< 0.01
Systolic blood pressure, mmHg	133 ± 25	129 ± 26	< 0.01
Diastolic blood pressure, mmHg	77 ± 17	76 ± 16	0.19
pH	7.43 ± 0.09	7.42 ± 0.11	0.02
PaCO_2_, mmHg	33 ± 7	33 ± 8	0.18
PaO_2_/FiO_2_, mmHg	167 ± 91	145 ± 76	< 0.01
SpO_2_, %	90 ± 7	87 ± 9	< 0.01
Variables collected after 1–2 h NIV			
GCS	14.8 ± 0.7	14.5 ± 1.5	< 0.01
Heart rate, beats/min	106 ± 21	111 ± 23	< 0.01
Respiratory rate, breaths/min	26 ± 6	31 ± 8	< 0.01
Systolic blood pressure, mmHg	129 ± 22	126 ± 24	0.03
Diastolic blood pressure, mmHg	74 ± 14	72 ± 14	0.02
pH	7.44 ± 0.07	7.41 ± 0.10	< 0.01
PaCO_2_, mmHg	33 ± 7	34 ± 12	0.17
PaO_2_/FiO_2_, mmHg	213 ± 92	156 ± 82	< 0.01
SpO_2_, %	97 ± 3	95 ± 5	< 0.01
Outcome			
Death in hospital	28 (4%)	245 (43%)	< 0.01
Length of ICU stay, days	7 (5–12)	8 (4–14)	0.92
Length of hospital stay, days	19 (12–28)	13 (6–24)	< 0.01

*NIV* noninvasive ventilation, *ARDS* acute respiratory distress syndrome, *SOFA* sequential organ failure assessment, *GCS* Glasgow coma scale

**Fig. 2:**
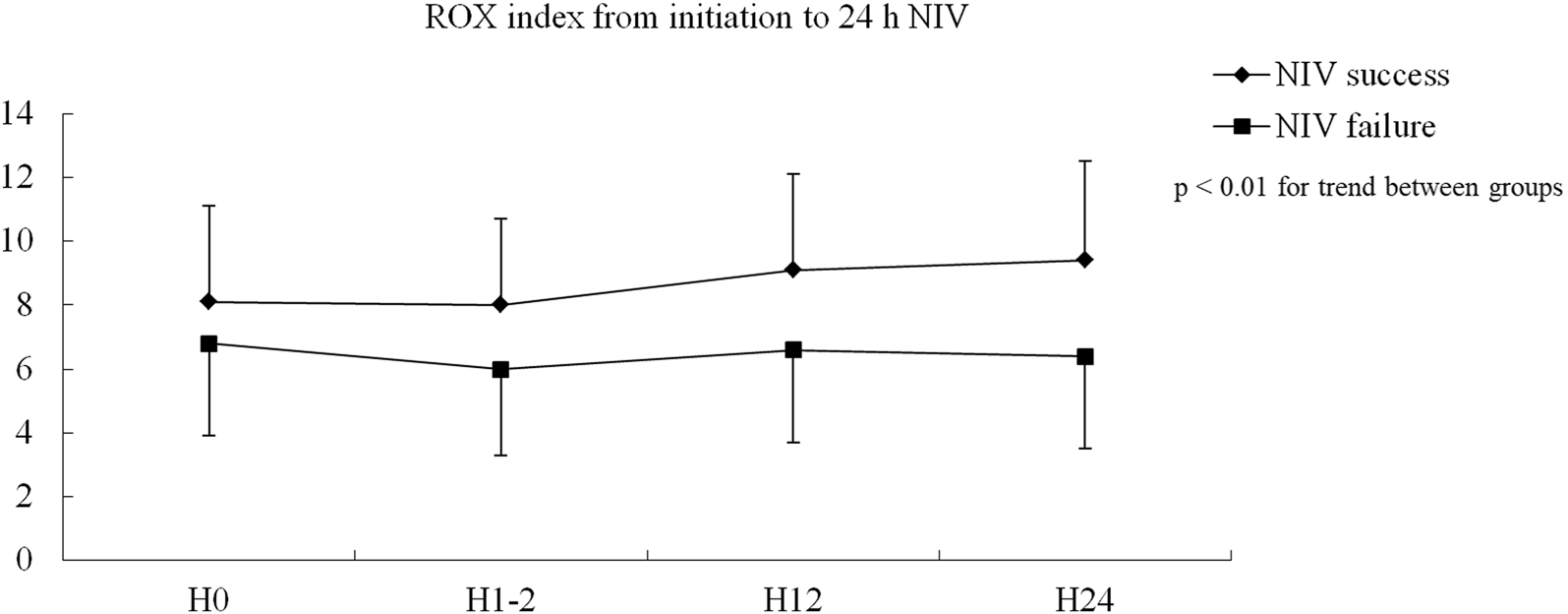
Comparisons in ROX index between patients with NIV success and failure. *ROX* SpO_2_/FiO_2_ to respiratory rate, *NIV* noninvasive ventilation

**Fig. 3:**
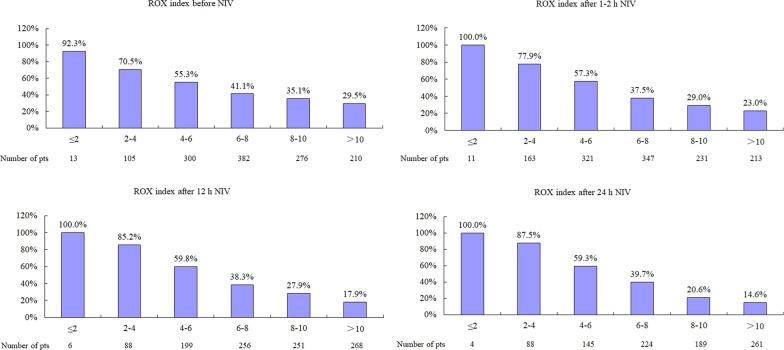
Rates of NIV failure in patients with different ROX index. *ROX* SpO_2_/FiO_2_ to respiratory rate, *NIV* noninvasive ventilation

In the NIV failure group, the ROX index was much lower than that in the successful NIV group when the index was calculated before NIV (Fig. 2). The rates of NIV failure were 92.3%, 70.5%, 55.3%, 41.1%, 35.1%, and 29.5% in patients with ROX index ≤ 2, 2–4, 4–6, 6–8, 8–10, and > 10, respectively (Fig. 3). Similar results were found when the ROX index was assessed after 1–2, 12, and 24 h NIV.

**Fig. 4:**
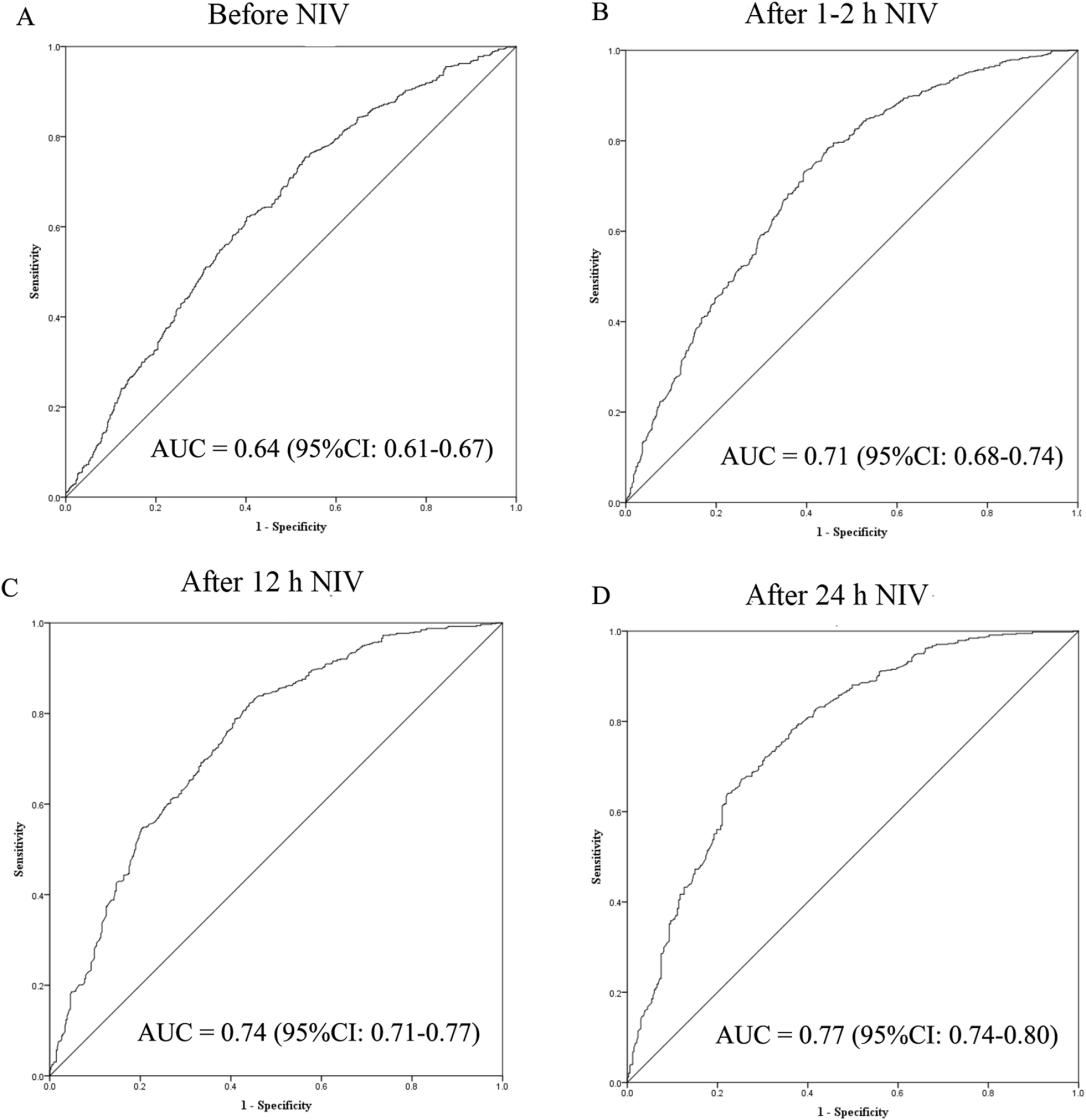
Predictive power of NIV failure tested by ROX index. *ROX* SpO_2_/FiO_2_ to respiratory rate, *AUC* area under the receiver operating characteristics curve, *NIV* noninvasive ventilation, *CI* confidence interval

Before NIV, the AUC was 0.64 (95% confidence interval [CI] 0.61–0.67) when the ROX index was used to predict NIV failure (Fig. 4). It increased to 0.71 (95% CI 0.68–0.74), 0.74 (0.71–0.77), and 0.77 (0.74–0.80) when the ROX index was assessed to predict NIV failure after 1–2, 12, and 24 h NIV, respectively. The sensitivity and specificity to predict NIV failure under different cutoff values of ROX index are presented in Table 2.

**Table 2:** Predictive power of NIV failure tested by different ROX index

Cutoff value	Probability of NIV failure (%)	Sensitivity (%)	Specificity (%)
After 1–2 h NIV, *N* = 1286			
2	75	100	2.1
4	65	95.0	24.3
6	50	75.9	56.7
8	37	45.7	79.6
10	25	22.8	91.4
After 12 h NIV, *N* = 1068			
2	79	100	1.4
4	67	97.9	19.5
6	53	85.7	48.6
8	37	61.5	71.6
10	24	33.7	88.5
After 24 h NIV, *N* = 911			
2	82	99.8	1.2
4	69	98.1	24.9
6	52	87.4	50.2
8	35	64.5	76.9
10	21	38.6	88.6

*NIV* noninvasive ventilation, *ROX* ratio of SpO_2_/FiO_2_ to respiratory rate

**Fig. 5:**
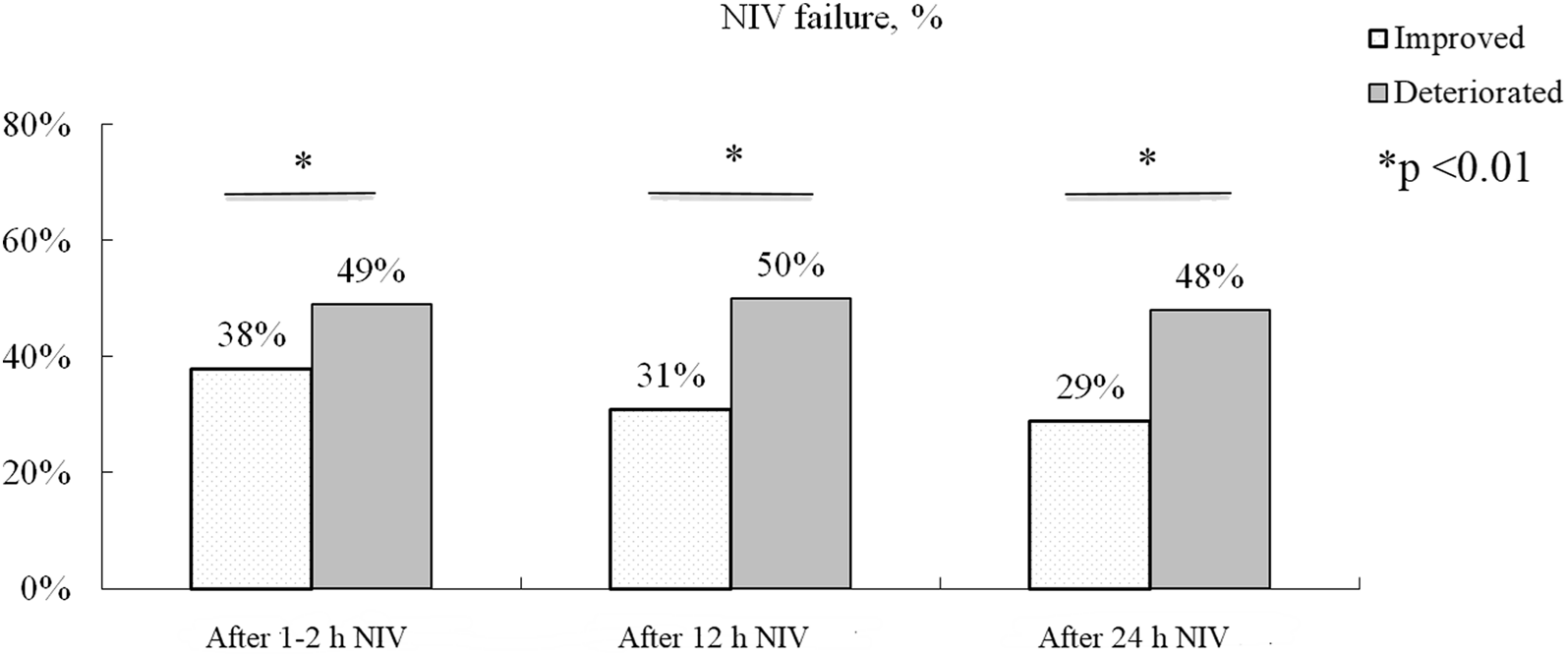
Rates of NIV failure in patients whose ROX index improved or deteriorated relative to values collected before NIV. *ROX* SpO_2_/FiO_2_ to respiratory rate, *NIV* noninvasive ventilation

After 1–2 h NIV, the probability of NIV failure was 25%, 50%, and 75% for the ROX index cutoff values of 2, 6, and 10, respectively. Patients were classified into low, moderate, high, and very high risk groups for ROX index values of > 10, 6–10, 2–6, and ≤ 2, respectively. The rates of NIV failure were 23%, 34.1%, 64.3%, and 100% in these respective groups when the ROX index was used to stratify patients after 1–2 h NIV. Compared to the ROX index before NIV, improved patients had lower rates of NIV failure than the deteriorated patients after 1–2, 12, and 24 h NIV (Fig. 5).

ROX index values had similar AUCs to PaO_2_/FiO_2_ when NIV failure was predicted within 24 h NIV (Table 3). Compared to PaO_2_/FiO_2_/RR, the ROX index also had similar AUCs before NIV and after 12 and 24 h NIV. Only after 1–2 h NIV was the AUC slightly higher in PaO_2_/FiO_2_/RR than that for the ROX index (0.74 vs. 0.71, *p* = 0.02).

**Table 3:** Comparisons in predictive power between ROX index, PaO_2_/FiO_2_, and PaO_2_/FiO_2_/RR

	AUC (95% CI)	*p*^a^	*p*^b^
Before NIV			
ROX index	0.64 (0.61–0.67)	0.10	0.47
PaO_2_/FiO_2_	0.61 (0.58–0.64)		
PaO_2_/FiO_2_/RR	0.65 (0.62–0.67)		
After 1–2 h NIV			
ROX index	0.71 (0.68–0.74)	0.84	0.02
PaO_2_/FiO_2_	0.71 (0.68–0.73)		
PaO_2_/FiO_2_/RR	0.74 (0.71–0.76)		
After 12 h NIV			
ROX index	0.74 (0.71–0.77)	0.14	0.41
PaO_2_/FiO_2_	0.72 (0.69–0.74)		
PaO_2_/FiO_2_/RR	0.75 (0.73–0.78)		
After 24 h NIV			
ROX index	0.77 (0.74–0.80)	0.26	0.47
PaO_2_/FiO_2_	0.75 (0.72–0.78)		
PaO_2_/FiO_2_/RR	0.78 (0.75–0.81)		

*NIV* noninvasive ventilation, *ROX* ratio of SpO_2_/FiO_2_ to respiratory rate, *AUC* area under the receiver operating characteristics curve, *CI* confidence interval, *RR* respiratory rate

^a^ROX index vs. PaO_2_/FiO_2_

^b^ROX index vs. PaO_2_/FiO_2_/RR

## Discussion

To the best of our knowledge, this is the largest study to explore the ROX index as a predictor for NIV failure in patients with de novo acute respiratory failure. The index had similar predictive power as PaO_2_/FiO_2_. It had a similar distinguishing power to PaO_2_/FiO_2_/RR in most cases. Three cutoff values (2, 6, and 10) were selected to classify patients into low, moderate, high, and very high risk for NIV failure.

The ROX index is mainly used to predict HFNC failure in patients with acute respiratory failure. In a classic study that focused on using the ROX index to predict HFNC failure, AUC was between 0.66 and 0.80 from initiation to 24 h HFNC [12]. In our study, AUC was similar, being between 0.64 and 0.77 for the prediction of NIV failure using the ROX index. This indicates that the predictive power of the ROX index for treatment failure in NIV patients was the same as that in HFNC patients. It is worth noting that the AUC was 0.64 when the index was used to predict NIV failure before NIV. It is difficult to predict NIV failure using only the ROX index at this time point. A combination of other risk factors can improve the predictive power. Using a comprehensive assessment tool such as the HACOR or updated HACOR score is another strategy to improve predictive accuracy [10, 17].

PaO_2_/FiO_2_ is associated with NIV failure [27]. Patients with lower PaO_2_/FiO_2_ values are more likely to experience NIV failure. It requires an arterial blood gas test to calculate PaO_2_/FiO_2_. However, this test is invasive and painful for patients. In addition, it is inconvenient to perform frequently. In contrast, measurement of the ROX index is noninvasive and can be performed at any time. Our study shows that the predictive power of the index for predicting NIV failure was similar to that of PaO_2_/FiO_2_. Therefore, ROX index can be served as an alternative method to predict NIV failure at any time.

Delayed intubation in NIV patients is associated with increased mortality [8, 9]. However, it is difficult to balance unnecessary and delayed intubation. The ROX index is a feasible assessment tool for predicting NIV failure at the bedside. It can be used to aid in decision-making, as it can stratify patients into low, moderate, high, and very high risk for NIV failure groups. In patients at high risk for NIV failure, NIV should be used cautiously. In those with very high risk for NIV failure, early intubation may be the best choice.

This study had several limitations. First, we only reported the ROX index within 24 h NIV. The predictive power of the index for use of NIV exceeding 24 h was not identified. Second, the use of NIV is a continuous process. Clinicians should assess the ROX index dynamically to avoid delayed intubation. Third, some physicians in this study may have used the ROX index in some patients at some point. This may have influenced their decision regarding intubation. As we did not record this issue, we are unable to report these data. Fourth, we only enrolled patients with de novo acute respiratory failure, due to the lack of clear evidence on the use of NIV in this population as recommended by the ERS/ATS guideline [18]. The index’s predictive power for NIV failure in other populations is unclear.

## Conclusions

The ROX index is convenient and reproducible. It has moderate power for predicting NIV failure in patients with de novo acute respiratory failure. Assessment of the ROX index at the bedside is encouraged when NIV is used.

## Data Availability

The data set used and/or analyzed during the current study is available from the corresponding author on reasonable request.

## Abbreviations

ROX: SpO_2_/FiO_2_ to respiratory rate
NIV: Noninvasive ventilation
AUC: Area under the receiver operating characteristic curve
HACOR: Heart rate, acidosis, consciousness, oxygenation, and respiratory rate
OR: Odds ratio
CI: Confidence interval
HFNC: High-flow nasal cannula
ARDS: Acute respiratory distress syndrome
SOFA: Sequential organ failure assessment
CT: Computed tomographic
ICU: Intensive care unit
GCS: Glasgow coma scale
RR: Respiratory rate
